# Effects of combined training on heart rate variability and cardiac function and structure in individuals with grade 1 obesity

**DOI:** 10.14814/phy2.70779

**Published:** 2026-02-24

**Authors:** Valéria Bonganha, Ivan Luiz Padilha Bonfante, Keryma Chaves da Silva Mateus, Arthur Fernandes Gáspari, Jamal Baracat, Guilherme De Rossi, Wilson Nadruz, Cláudia Regina Cavaglieri, Mara Patricia Traina Chacon‐Mikahil

**Affiliations:** ^1^ Laboratory of Exercise Physiology, Faculty of Physical Education Universidade Estadual de Campinas (UNICAMP) Campinas SP Brazil; ^2^ Postdoctoral Researcher Program (PPPD) Universidade Estadual de Campinas (UNICAMP) Campinas SP Brazil; ^3^ Brazilian Sport Climbing Confederation São Paulo SP Brazil; ^4^ Faculty of Medical Sciences Universidade Estadual de Campinas (UNICAMP) Campinas SP Brazil

**Keywords:** abdominal fat, combined training, echocardiogram, heart rate variability

## Abstract

Excess body fat, particularly in the abdominal region, increases the risk of cardiovascular disease. Conversely, aerobic training can induce beneficial effects on heart rate variability (HRV), as well as on cardiac structure and function, along with favorable changes in body composition. However, the interrelationship between changes in HRV, cardiac parameters, and adiposity induced by combined training (strength training followed by aerobic training; CT) in obese individuals remains unclear. Therefore, the present study evaluated the effects of 24 weeks of CT on body composition, physical fitness, ultrasonography‐based abdominal fat estimation, echocardiographic parameters, and HRV in obese individuals without dietary modifications. Twenty‐eight obese middle‐aged men participated in the study: 16 individuals were part of the combined training group (CTG), performing resistance and aerobic training three times per week (~60 min per session), and 12 individuals comprised the control group (CG), who did not engage in any structured training program. Following the intervention, improvements were observed in echocardiographic functional variables, including systolic myocardial velocity, early diastolic myocardial velocity, the ratio of early to late diastolic myocardial velocity, peak early diastolic filling velocity, and the ratio of peak early to late diastolic filling. Additionally, significant enhancements in HRV parameters (RR interval, RMSSD, and low‐ and high‐frequency components) were detected. Concomitantly, reductions in visceral adiposity were documented. Furthermore, significant correlations were observed between adaptations on cardiac functional and HRV indices with clinical variables. Collectively, these findings suggest that CT promotes favorable cardiac functional adaptations that are closely associated with enhanced HRV, while simultaneously reducing visceral adiposity and improving clinical variables.

## 1 INTRODUCTION

Obesity represents one of the most critical public health challenges worldwide, given its strong association with the development and progression of cardiovascular diseases. Notably, even in the absence of overt comorbidities, obese individuals frequently exhibit alterations in cardiac structure and function, as well as impairments in autonomic modulation (Grassi et al., 2019; Lembo et al., 2024). Excess body fat, particularly visceral adiposity, can profoundly affect cardiac morphology, leading to left ventricular (LV) chamber enlargement due to chronic overload. This process may culminate in pathological eccentric hypertrophy, reduced diastolic compliance, and elevated LV filling pressures (De Pergola et al., 2013). Beyond LV remodeling, additional structural alterations have been reported in the left atrium, interventricular wall, cardiac valves, and aortic dimensions (Alpert et al., 2016).

These structural abnormalities are often accompanied by functional derangements in cardiac cycle dynamics, blood volume, heart rate, cardiac output (CO), and systemic perfusion, which collectively contribute to elevated cardiac, pulmonary, and systemic pressures (Alpert et al., 2016; De Pergola et al., 2013).

The pathophysiological mechanisms underlying these structural and functional changes are multifactorial and involve a complex interplay of chronic low‐grade systemic inflammation—primarily driven by visceral and ectopic fat accumulation—along with neuroendocrine and metabolic dysregulation, heightened activation of the renin–angiotensin–aldosterone system, and profound alterations in autonomic nervous system regulation (Alpert et al., 2016). Specifically, obesity is characterized by increased sympathetic nervous system (SNS) activity and concomitant reductions in parasympathetic nervous system (PNS) activity (Grassi et al., 2019; Shaffer & Ginsberg, 2017), a profile that markedly increases cardiometabolic risk and accelerates the development of obesity‐related comorbidities (Alpert et al., 2016; Grassi et al., 2019; Shaffer & Ginsberg, 2017).

Among the available non‐pharmacological interventions, physical training has emerged as an important strategy for obesity management and cardiovascular disease prevention (Garber et al., 2011). Aerobic training, in particular, has demonstrated robust benefits for cardiovascular health and autonomic modulation (Soltani et al., 2025). Resistance training may serve as a valuable adjunct by providing additional cardiovascular benefits and inducing favorable adaptations in body composition and overall health status (Garber et al., 2011; Saunders et al., 2022). Consequently, combined training (CT)—which integrates resistance and aerobic exercise modalities—has been strongly recommended as an optimal strategy for health maintenance and improvement (Garber et al., 2011). Evidence suggests that CT improves low‐grade inflammation, insulin resistance, and metabolic profiles in individuals with chronic non‐communicable diseases (Bonfante et al., 2022; Brunelli et al., 2015; Duft et al., 2024), thereby contributing to enhanced autonomic function and favorable cardiac remodeling.

Although aerobic training is widely recognized for its direct effects on autonomic modulation and cardiac structure and function, and resistance training is known to confer indirect cardiovascular benefits, uncertainties remain regarding the specific effects of CT on these outcomes. These uncertainties relate to potential interference between training modalities, variations in training volume, intensity, frequency, and duration, the sequencing of exercise modalities within training sessions, population‐specific responses (e.g., individuals with excess adiposity), and the complex interplay between cardiac and autonomic adaptations (Murray et al., 2022; Yang et al., 2024).

Despite this body of evidence, the interrelationship between CT‐induced adaptations in cardiac function, heart rate variability (HRV) as a marker of autonomic modulation, and abdominal fat remains insufficiently elucidated, particularly in obese populations. Thus, the present study aimed to evaluate the effects of a 24‐week CT intervention on abdominal fat, HRV, and echocardiographic indices of cardiac structure and function in men with grade 1 obesity, in the absence of dietary modification.

## 2 METHODS

### 2.1 Subjects

This article presents the secondary analyses (HRV, cardiac function and structure, and abdominal fat) of the controlled trial entitled “Exercise training effects on biochemical, metabolic, physical fitness, body composition and hemodynamic markers” (ACTRN12615001000594). The primary results of this trial were previously published and evaluated the effects of exercise training protocols on physical fitness and metabolic/inflammatory markers in this population (Bonfante et al., 2017; Brunelli et al., 2015; Duft et al., 2017). The study was approved by the Research Ethics Committee of the *Universidade Estadual de Campinas* and was based on the principles of the Declaration of Helsinki. All volunteers were informed about the study and signed an informed consent form.

The present study enrolled 28 middle‐aged males who had not engaged in regular exercise programs during the previous 12 months according to the Baecke Habitual Physical Activity Questionnaire and were classified as physically inactive and obese (body mass index (BMI) between 30 and 35 kg/m^2^) (Brunelli et al., 2015). As exclusion criteria, the subjects should be free of coronary artery disease, severe hypertension, diabetes mellitus, chronic obstructive pulmonary disease, limiting osteoarticular diseases, or the use of any medication that could interfere in the physiological responses of testing or training. For the combined training group (CTG), discontinuation criteria included attendance below 85% of training sessions and/or missing more than two consecutive sessions. Additionally, only subjects with available heart rate variability and/or cardiac function and structure data were included in the final analysis.

**FIGURE 1:**
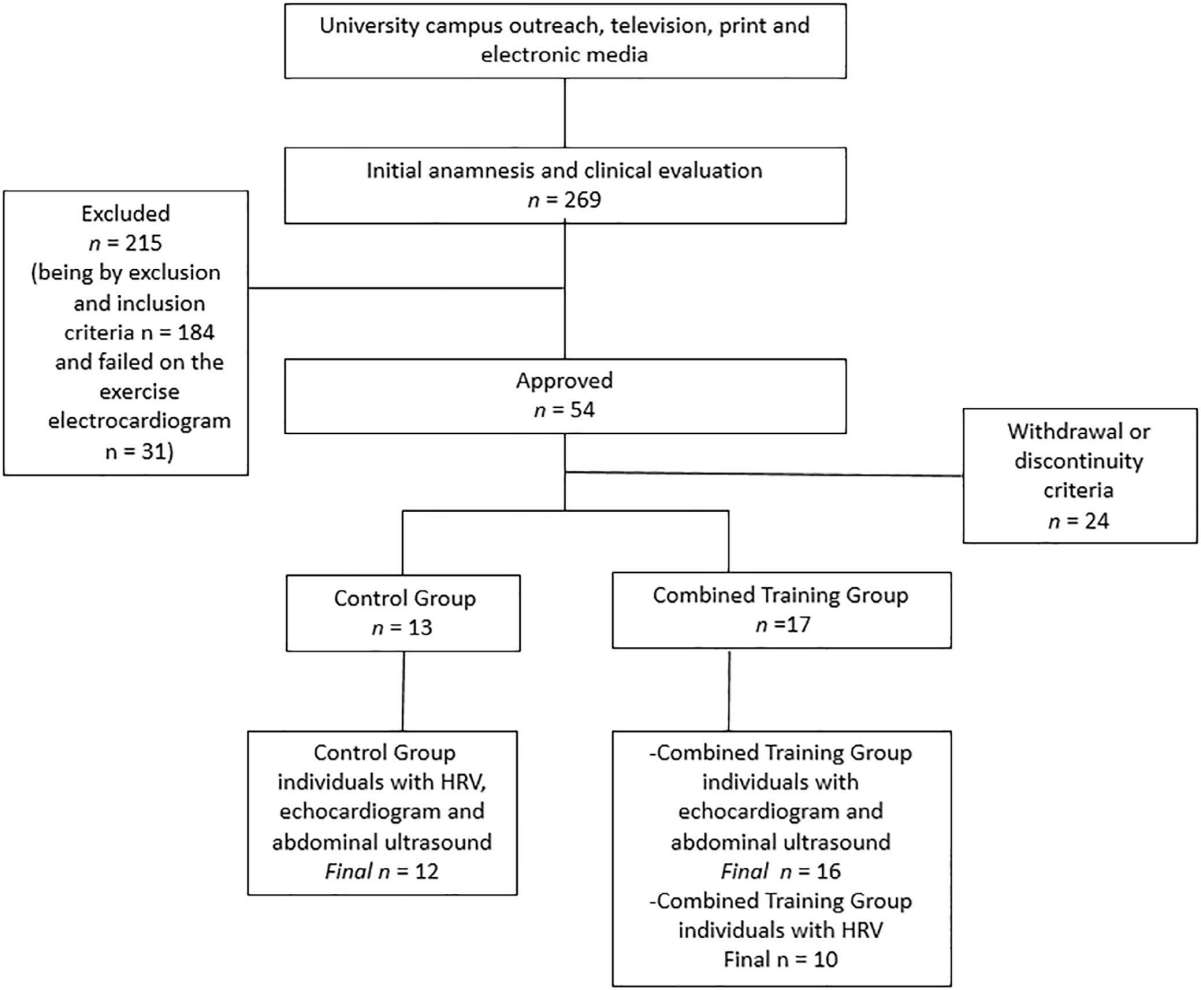
Flowchart.

In summary, following recruitment through advertisements on the university campus and in local media, 269 obese individuals underwent an initial interview. Of these, 215 were excluded or deemed ineligible during the clinical evaluation or electrocardiographic (ECG) screening. Consequently, 54 participants were approved, randomized, and initiated the study, being allocated to either the combined training group (CTG) or the control group (CG). However, 24 individuals subsequently dropped out or were excluded according to predefined discontinuity criteria. Thus, 30 participants were included in the primary study analyses (CTG, *n* = 17; CG, *n* = 13). The present study, however, includes different sample sizes for specific outcomes due to technical limitations. Specifically, two participants (one from each group) did not undergo pre‐ and post‐intervention echocardiographic and abdominal ultrasound assessments. Additionally, seven participants from the CTG and one participant from the CG did not complete post‐intervention HRV assessments. As a result, the final sample sizes for each assessment were as follows: for echocardiographic and abdominal ultrasound evaluations, CG (*n* = 12) and CTG (*n* = 16); for HRV analyses, CG (*n* = 12) and CTG (*n* = 10). All participants included in the HRV analyses also underwent echocardiographic and abdominal ultrasound assessments. The participant flow is illustrated in Figure 1.

### 2.2 Study design

In addition to HRV, echocardiogram and abdominal ultrasound, the CTG and CG groups performed clinical (hemodynamics, anthropometry, body composition by body density using the Siri equation and skin‐fold procedure by skin‐fold caliper; and blood sampling) and functional (cardiorespiratory and maximal‐strength assessments) evaluations before and after experimental period. To ensure that the dietary pattern was maintained (in addition to the routine guidance for its maintenance), a standardized food record (FR) was also applied to both groups during the pre‐and post‐experimental period to obtain the total caloric intake and the proportion of carbohydrates, lipids and proteins consumed. More information about anthropometric assessments, body composition, nutritional assessment and markers assessed in the blood are shown in the primary study (Brunelli et al., 2015).

All subjects were previously familiarized with the functional evaluation protocols and equipment, and those in the CTG also underwent familiarization with the training protocol. All evaluations were carried out in controlled environmental temperature (23°C), relative humidity, and atmospheric pressure. Post‐evaluations were conducted at least 72 h after the final training session, with appropriate intervals between assessments. Additionally, participants were advised to abstain from alcohol for 24 h and caffeine or other stimulants for 12 h prior to each test.

Participants were formally monitored monthly and reminded to maintain their eating habits and physical activity, as well as report changes in other aspects of daily life and routine. The CTG underwent 24 weeks of combined training, while CG did not participate in any training protocol and was followed up monthly by telephone during the experimental period. After the second and fourth months, fitness levels were measured for training settings.

### 2.3 Evaluations

#### 2.3.1 Anthropometry, body composition, functional variables, and nutritional assessment

Body weight and height were measured with a calibrated manual scale (Filizola, São Paulo, Brazil) and with a wall‐mounted stadiometer. BMI was calculated from the weight and height values. Waist circumference (WC) was measured by the commonly established anatomical landmarks. Body composition was evaluated by subcutaneous skinfold thickness in triplicate, at the chest, abdomen, and thigh using a skinfold caliper. Body density was estimated using the equation of Jackson and Pollock, and percentage body fat was estimated from body density using Siri's Equation (Siri, 1961).

The cardiorespiratory assessment was performed using a progressive effort protocol on a treadmill, with gas analyzer (CPX Ultima, MedGraphics, USA). The maximum oxygen consumption (VO_2peak_) was established by the average values of the last 30 s of the test (Wasserman et al., 1973). The maximum repetition test (1RM) was applied for bench press and leg press exercises (Brunelli et al., 2015).

It was requested that the volunteers did not change their diet. To ensure that food behavior was being followed, the volunteers were routinely alerted to this aspect and even filled out FR. The FR were analyzed semi‐quantitatively delimiting the macronutrient and total caloric intake (Brunelli et al., 2015).

#### 2.3.2 Ultrasonography‐based fat estimation

A single operator, who was blinded to the treatment, measured abdominal fat thickness in triplicate using echography. The patient was in the supine position and the probe‐holding belt was worn around the patient's waist, centered at the umbilicus in order to standardize the following image acquisition and distance measurement steps. The clinician then identified the abdominal aorta in ultrasound images taken from three different positions. A 3.5‐MHz convex transducer was placed without compression in the xypho‐umbilical line right above aortic bifurcation (above the navel). Subcutaneous (SF) and visceral fat (VF) thicknesses corresponded to the distances, respectively, between the skin‐fat and fat‐muscle interfaces and between the internal face of the muscle and the aorta posterior wall (Lima et al., 2013).

#### 2.3.3 Framingham risk score

The Framingham Risk Score was used to estimate the risk of cardiovascular disease over 10 years. This score uses the following risk factors: age, sex, total cholesterol, high‐density lipoprotein (HDL), blood pressure, smoking, and history of diabetes (D'Agostino Sr. et al., 2008). Total cholesterol (enzyme‐trind method) and HDL cholesterol (accelerator‐selective detergent method) were evaluated with blood collected after 12 h of fasting.

#### 2.3.4 Measurement and analysis of HRV

The HRV measures were carried out between 7:00 a.m. and 12:00 a.m. in a quiet room with stable temperature, and HR was continuously recorded for 20 min at supine position (Polar S810i, Kempele, Finland). The validity of this instrument used to assess HRV data was described previously (Gamelin et al., 2006; Nunan et al., 2009). Time series data (two sets of 5 min length) were processed using Kubios HRV Analysis software (MATLAB, version 2 beta, Kuopio, Finland) and was used the average of the two consecutive windows, with 5 min of length in middle of the sample (Gamelin et al., 2006; Nunan et al., 2009). The participants were instructed to avoid alcohol and/or caffeinated beverages and exercise and/or vigorous activity for at least 24 and 72 h before the evaluation, respectively.

HRV was analyzed using both time and frequency domains. Time domain analysis included: R‐R interval mean (R‐Ri); the NN interval standard deviation (SDNN); and the root mean square of the squares of the differences between successive R‐Ri (rMSSD) in milliseconds. Frequency domain analysis comprised: Low frequency (LF‐ms^2^), high frequency (HF‐ms^2^); and LF/HF (Shaffer & Ginsberg, 2017). Rest heart rate (RHR), systolic blood pressure (SBP), and diastolic blood pressure (DBP) were measured on the test day (after 20 min of rest in a supine position) (Polito et al., 2007).

#### 2.3.5 Echocardiography

Echocardiography was performed by a skilled physician using the Vivid 3 (General Electric, Milwaukee, WI, USA), equipped with a 2.5 MHz transducer, as previously described (Matos‐Souza et al., 2011). The subjects positioned themselves in lateral decubitus and the stretcher remained slightly inclined. Left ventricular end‐diastolic and end‐systolic volume were measure from the acquisition of 2D images in M‐mode, according to the recommendations of the American Society of Echocardiography (Lang et al., 2006). The relative wall thickness (RWT) of the LV was calculate as twice the thickness of the posterior wall (PW) divided by the left ventricular end‐diastolic diameter. Left ventricular mass (LVM) and ejection fraction (EF) were calculated by the device. LV mass was calculated according to the formula: (1.04×(LV end‐diastolic diameter + septal thickness + posterior wall thickness)3‐(LV end diastolic diameter)) 3–13.6 g (Devereux & Reichek, 1977). The Teichholz formula was used to estimate the left ventricular ejection fraction (LVEF) from the left ventricular internal dimension at end‐diastole (LVIDd) (Lang et al., 2006). The mitral flow velocity was examined with pulsed Doppler and the following indexes were evaluated: peak velocity of early diastolic filling (E), peak velocity of late diastolic filling (A), E/A–ratio of peak early and late diastolic filling. Tissue Doppler imaging was use to evaluate the septum and lateral ventricular walls, as previously described (Matos‐Souza et al., 2011). The systolic (S′), early diastolic (E'), late diastolic (A') myocardial velocity and ratio of early and late diastolic myocardial velocity (E'/A') were analyzed from 3 consecutive beats. The stroke volume (SV) was obtained from the subtraction of the end‐diastolic volume by the end‐systolic volume. The cardiac output (CO) was calculated as product as stoke volume × heart rate. All examinations were performed by the same professional.

#### 2.3.6 Combined training protocol

The CT was composed of resistance and endurance exercises in the same session, respectively. The participants performed three weekly sessions on alternate days, during 24 weeks. The training program was structured into three eight‐week phases with a gradual increase in workloads. Endurance training sessions maintained a constant duration, with intensities ranging from 55% to 85% of VO_2_peak, performed through walking or running on a track, alternating periods below the ventilatory threshold (VT), at VT, and above VT up to below the respiratory compensation point (RCP).

Resistance training included exercises for the lower and upper body, performed in three sets of 6–10 maximum repetitions. Each session lasted approximately 60 min, beginning with resistance training and followed by aerobic training.

Aerobic training intensities were determined based on maximal treadmill tests, which were repeated every 8 weeks to adjust workloads. Resistance training loads were adjusted weekly according to performance, with progressive increases in weight. Every 8 weeks, the number of repetitions per set was reduced, while training loads and rest intervals were increased. Additional details regarding the training protocol are provided in the primary study (Brunelli et al., 2015).

#### 2.3.7 Statistical analysis

Initially, the Kolmogorov–Smirnov test was applied to assess data normality. Between‐group differences in pre‐ moment and pre‐post‐intervention percentage changes (Δ%) were analyzed using independent Student's *t*‐tests. A two‐way analysis of variance (ANOVA) with repeated measures was employed. Only when a significant main effect or interaction (time × group) was detected, Tukey's post hoc test was applied. For the echocardiographic E′ variable, covariance analysis (ANCOVA) was performed due to baseline differences between groups. Pearson's correlation coefficients were calculated to assess associations between Δ% changes in HRV and echocardiographic variables and metabolic and functional parameters (variables presented in Table 1 plus glucose and lipid profile) in the CTG. The level of significance used was *p* ≤ 0.05. *p*‐values between 0.06 and 0.09 were also reported—particularly in Tukey's post hoc analyses—due to their potential clinical relevance, especially in studies with small sample sizes (Thiese et al., 2016). All statistical analyses were performed using Statistica software (version 6.0). Within group effect sizes were calculated using Cohen's d. Cohen's d interpretation was: <0.5 = small; between 0.5 and 0.79 = medium; and >0.8 = high.

## 3 RESULTS

To improve the understanding of the present results, it is important to mention that the results occurred in parallel with the improvement in glycemic control, lipid profile, body composition, physical fitness, and inflammatory markers. No significant changes were observed in food behavior (data presented only in the primary studies) (Bonfante et al., 2017; Brunelli et al., 2015; Duft et al., 2017).

However, as the number of subjects here analyzed is different from the primary studies, we performed statistical analysis of some body composition and functional capabilities. These pre‐post and Δ% data are presented in Table 1. The CTG decreased WC (Tukey *p* = 0.02; Δ% *t*‐test *p* = 0.01), body fat (Tukey *p* = 0.0001; Δ% *t*‐test *p* = 0.003); VF (Tukey *p* = 0.04; Δ% *t*‐test *p* = 0.04), DBP (only by Δ% *t*‐test *p* = 0.01) and RHR (Δ% *t*‐test *p* = 0.02). The CTG also increases free fat mass (Tukey *p* < 0.0001; Δ% *t*‐test *p* = 0.004), VO2 (Tukey *p* = 0.001; Δ% *t*‐test *p* = 0.01) and body strength (Tukey *p* < 0.0001; Δ% *t*‐test *p* < 0.0001). For the Framingham risk score, although the ANOVA identified a group × time difference (*p* = 0.03), no differences were observed by post hoc analysis, while in the Δ% there is only a trend of difference between groups (CTG: 4.17, CG: 6.35; *p* = 0.07) and in pre‐pos CTG. No changes were observed in BMI, subcutaneous fat and systolic blood pressure.

**TABLE 1:** Pre‐post body composition, clinical characteristics and functional capabilities.

	Control				Combined training				*p* ANOVA	*F* value
	Pre	Post	∆%	E.S.	Pre	Post	∆%	E.S.	G × T	G × T
Age (years)	49 ± 6	‐	‐	‐	50 ± 6	‐	‐	‐	‐	‐
BMI (kg/m^2^)	31.03 ± 1.55	31.24 ± 2.01	0.62	0.11	30.90 ± 1.70	30.58 ± 1.64	−1.00	0.19	0.08	3.41
WC (cm)	101.88 ± 3.43	102.97 ± 5.33	0.96	0.24	102.54 ± 5.02	100.16 ± 5.17^a^	−2.31^b^	0.46	0.01	6.73
BF (kg)	32.89 ± 7.66	32.55 ± 7.10	−0.51	0.04	33.67 ± 7.96	26.05 ± 7.66^a^	−22.34^b^	0.97	0.001	9.94
FFM (kg)	62.81 ± 5.70	64.24 ± 5.50	2.21	0.25	59.69 ± 3.72	66.43 ± 5.09^a^	11.42^b^	1.51	0.001	9.14
SF (mm)	25.46 ± 8.13	24.85 ± 7.32	−0.71	0.07	24.75 ± 6.85	21.44 ± 8.95	−13.19	0.41	0.26	1.30
VF (mm)	80.74 ± 15.33	78.26 ± 22.23	−3.64	0.12	81.33 ± 19.09	66.95 ± 11.76^a^	−16.26^b^	0.90	0.04	4.18
SBP (mm Hg)	137.50 ± 10.18	143.42 ± 15.04	4.40	0.46	137.44 ± 14.50	139.13 ± 13.41	1.58	0.12	0.34	0.93
DBP (mm Hg)	92.08 ± 5.62	94.92 ± 5.62	3.08	0.50	91.25 ± 10.01	89.56 ± 7.82	−1.51^b^	0.18	0.09	3.09
HR (beats/min)	72.33 ± 10.17	76.50 ± 11.01	6.24	0.29	72.94 ± 12.74	68.56 ± 10.48	−4.96^b^	0.37	0.02	6.13
FR score (%)	10.21 ± 4.11	11.27 ± 5.82	6.35	0.21	10.91 ± 4.29	9.97 ± 3.35	−4.17	0.24	0.03	6.28
VO_2peak_ (mL/kg/min)	28.18 ±4.64	28.80 ± 4.23	2.21	0.13	28.13 ± 4.25	31.09 ± 4.25^a^	10.43^b^	0.69	0.01	7.75
Body Strength (kg)	426.75 ± 89.81	429.88 ±87.92	0.93	0.03	401.40 ± 71.49	485.73 ± 71.49^a^	22.85^b^	1.17	<0.001	33.02

*Note*: Control group *n* = 12. Combined training group *n* = 16.

Abbreviations: BF, body fat; BMI, body mass index; DBP, diastolic blood pressure; E.S., effect size; FFM, free fat mass; FR score, framingham score; G × T = group × time ANOVA effect; HR, heart rate; SBP, systolic blood pressure; SF, subcutaneous fat; VF, visceral fat; VO_2peak_, peak oxygen consumption; WC, waist circumference.

^a^ Difference pre‐post by Tukey post hoc (*p* ≤ 0.05).

^b^ Difference between ∆% by independent *t*‐test (*p* ≤ 0.05). Observation: Data of table previously published in Brunelli et al. (2015) using a different number of subjects in each group.

Table 2 shows the pre‐post data of cardiac structure. No significant differences were observed for any of the studied variables.

**TABLE 2:** Pre‐post cardiac structure measurements.

	Control				Combined training				*p* ANOVA	*F* value
	Pre	Post	∆%	E.S.	Pre	Post	∆%	E.S.	G × T	G × T
LAD (mm)	37.69 ± 2.91	38.70 ± 2.00	3.31	0.48	38.08 ± 2.31	38.10 ± 3.41	0.13	0.01	0.42	0.66
LVEDV (mL)	130.85 ± 12.25	124.29 ± 15.78	−5.82	0.48	126.37 ± 15.03	128.97 ± 16.56	2.53	0.16	0.08	2.69
LVESV (mL)	43.61 ± 7.71	41.56 ± 7.33	−4.06	0.27	42.85 ± 7.17	45.09 ± 7.97	4.09	0.30	0.18	1.83
IS (mm)	8.23 ± 1.22	8.98 ± 0.92	10.61	0.69	8.75 ± 0.87	9.16 ± 1.04	5.23	0.42	0.39	0.39
PWT (mm)	8.37 ± 0.96	8.76 ± 0.74	5.42	0.45	8.45 ± 0.86	8.82 ± 1.20	4.74	0.35	0.95	0.01
RWT (mm)	0.32 ± 0.03	0.34 ± 0.03	7.50	0.31	0.33 ± 0.03	0.34 ± 0.04	3.85	0.28	0.45	0.56
LVM (g)	192.39 ± 35.80	202.23 ± 32.2	5.46	0.28	198.65 ± 33.49	211.26 ± 48.95	6.50	0.30	0.82	0.05
LVMI (g m^−2^)	72.76 ± 13.85	75.69 ± 12.35	5.25	0.22	76.20 ± 12.76	81.60 ± 17.55	7.32	0.35	0.58	0.30

*Note*: Control group *n* = 12. Combined training group *n* = 16.

Abbreviations: E.S., effect size; G × T, group × time ANOVA effect; IS, interventricular septum; LAD, left atrium diameter; LVED, left ventricular end‐diastolic volume; LVESV, left ventricular end‐systolic volume; LVM, left ventricular mass; LVMI, left ventricular mass index; PWT, posterior wall thickness; RWT, relative wall thickness.

The variables related to cardiac function are presented in Table 3. The CTG increased S′ (Tukey *p* = 0.06; Δ% *t*‐test *p* = 0.03), E (Tukey *p* = 0.08; Δ% *t*‐test *p* = 0.006), E'/A' (only by Δ% *t*‐test *p* = 0.03) and E/A (Tukey *p* = 0.08; Δ% *t*‐test *p* = 0.006). The diastolic variable E' that presented significant difference at the pre moment (*p* = 0.01), therefore ANCOVA was applied, which showed that the training or the time as control promoted opposing alterations independent of the pre values, it was observe an increase for the CTG and a reduction for the GC (ANCOVA *p* = 0.008; Δ% *t*‐test *p* < 0.001). No other significant differences were observed (Table 3).

**TABLE 3:** Pre‐post cardiac function measurements.

	Control				Combined training				*p* ANOVA	*F* value
	Pre	Post	∆%	E.S.	Pre	Post	∆%	E.S.	G × T	G × T
SV (mL)	86.75 ± 11.24	82.74 ± 10.58	−4.58	0.36	83.52 ± 10.30	86.06 ± 8.72	3.33	0.26	0.12	2.49
CO (L/min)	5.78 ± 2.04	6.27 ± 0.78	0.88	0.31	6.01 ± 1.17	5.75 ± 1.18	−3.83	0.22	0.45	0.58
EF (%)	66.69 ± 5.13	67.32 ± 3.53	2.01	0.14	65.47 ± 3.69	65.31 ± 3.84	−0.32	0.04	0.44	0.59
S′ (cm s^−1^)	12.52 ± 1.60	11.77 ± 1.98	−5.5	0.41	11.59 ± 3.14	12.83 ± 3.18	12.14^d^	0.41	0.04	4.35
E' (cm s^−1^)	14.40 ± 2.41	12.03 ± 3.14^b^, ^c^	−16.87	0.84	11.38 ± 3.18	13.22 ± 2.31^c^	19.43^d^	0.66	^a^	^a^
A' (cm s^−1^)	13.07 ± 2.49	11.79 ± 1.80	−6.83	0.58	11.91 ± 1.97	11.93 ± 2.24	1.41	0.01	0.19	1.75
E (cm s^−1^)	78.28 ± 16.47	69.48 ± 10.50	−10.13	0.63	66.19 ± 16.16	75.81 ± 17.47	15.67^d^	0.57	< 0.01	11.32
A (cm s^−1^)	62.92 ± 13.16	65.02 ± 10.95	10.77	0.17	64.38 ±13.89	62.95 ± 11.42	−3.61	0.11	0.16	2.11
E'/A'(cm s^−1^)	1.11 ± 0.14	1.05 ± 0.31	−6.16	0.24	0.98 ± 0.29	1.14 ± 1.28	21.33^d^	0.18	0.06	3.66
E/A (cm s^−1^)	1.26 ± 0.23	1.09 ± 0.24	−16.42	0.72	1.08 ± 0.38	1.25 ± 0.35	24.11^d^	0.46	0.01	8.82
E/E' (cm s^−1^)	5.42 ± 0.96	5.95 ± 0.94	7.98	0.55	5.85 ± 0.98	5.87 ± 1.24	1.42	0.02	0.24	1.40

*Note*: Control group *n* = 12. Combined training group *n* = 16.

Abbreviations: A', late diastolic myocardial velocities; A, peak velocity of late diastolic filling; CO, cardiac output; E', early diastolic myocardial velocities; E, peak velocity of early diastolic filling; E'/A', ratio of early and late diastolic myocardial velocities; E/A, ratio of peak early and late diastolic filling; E/E', ratio of peak early inflow velocity and longitudinal peak early diastolic velocities; EF, ejection fraction; E.S., effect size; G × T = group × time ANOVA effect; S′, systolic myocardial velocity; SV, stroke volume.

^a^ ANCOVA applied.

^b^ Difference pre‐post by Tukey post hoc (*p* ≤ 0.05).

^c^ Difference pre‐post between groups by ANCOVA (*p* ≤ 0.05).

^d^ Difference between ∆% by independent *t*‐test (*p* ≤ 0.05).

Table 4 presents the results of the HRV variables. Although RR interval, RMSSD and LF demonstrated significant group × time interaction in ANOVA (*p* < 0.05), only a trend toward a pre‐to post difference was observed in RR interval in the CTG (Tukey *p* = 0.09). However, when comparing the Δ%, a difference is observed for the three variables RR (*t‐*test *p* = 0.03), RMSSD (*t‐*test *p* = 0.01) and LF (*t‐*test *p* = 0.03), and in all of them the CTG shows an increase in the means. For SDNN and HF, only a trend of significance is observed in ANOVA (*p* = 0.06), while in the comparison between the Δ%, there is a significant difference for HF (*t‐*test *p* = 0.01), while for SDNN there is only a tendency of difference (*t*‐test *p* = 0.07). No differences were observed in the LF/HF.

**TABLE 4:** Pre‐post heart rate variability measurements.

	Control				Combined training				*p* ANOVA	*F* value
	Pre	Post	∆%	E.S.	Pre	Post	∆%	E.S.	G × T	G × T
RRi (mean)	895.84 ± 124.38	886.23 ± 113.86	−1.97	0.08	885.51 ± 91.82	945.36 ± 98.33	7.03^a^	0.62	0.03	4.96
SDNN (ms)	28.93 ± 12.99	25.97 ± 11.84	−6.10	0.23	29.70 ± 11.61	40.89 ± 6.85	33.54	1.17	0.06	3.77
RMSSD (ms)	24.03 ± 15.97	19.75 ± 13.16	−19.48	0.29	19.30 ± 8.56	26.17 ± 10.91	25.18^a^	0.70	0.04	4.43
LF (ms^2^)	481.08 ± 452.60	486.09 ± 576.97	4.00	0.01	537.89 ± 408.79	767.00 ± 461.13	72.98^a^	0.52	0.02	5.44
HF (ms^2^)	304.18 ± 443.55	262.45 ± 319.68	−24.76	0.10	155.80 ± 110.49	276.44 ± 243.72	68.30^a^	0.63	0.06	3.94
LF/HF	2.21 ± 1.26	2.31 ± 2.18	2.26	0.05	3.53 ± 2.52	3.15 ± 1.64	−7.74	0.18	0.38	0.80

*Note*: Control group *n* = 12. Combined training group *n* = 10.

Abbreviations: E.S., effect size; G × T, group × time ANOVA effect (*p* ≤ 0.05); HF, High frequency; LF, low frequency; LF/HF, LF/HF ratio; RMSSD, the root mean square of the squares of the differences between successive R‐Ri; RRi mean, RR interval mean; SDNN, NN interval standard deviation.

^a^ Difference between ∆% by independent *t*‐test (*p ≤* 0.05).

Of the 17 significant results (ANOVA/post hoc and/or Δ%) reported in Tables 1, 2, 3, 4, 11 exhibited medium to high or very high effect size in the CTG, were near a medium effect size (WC, S′, E/A), while only three demonstrated low effect sizes (RHR, DBP, and E′/A′). These findings corroborate the results of the inferential statistics, particularly when considering the antagonistic changes observed in the CG for most variables. Notably, a very high effect size was observed for SDNN in the CTG.

**FIGURE 2:**
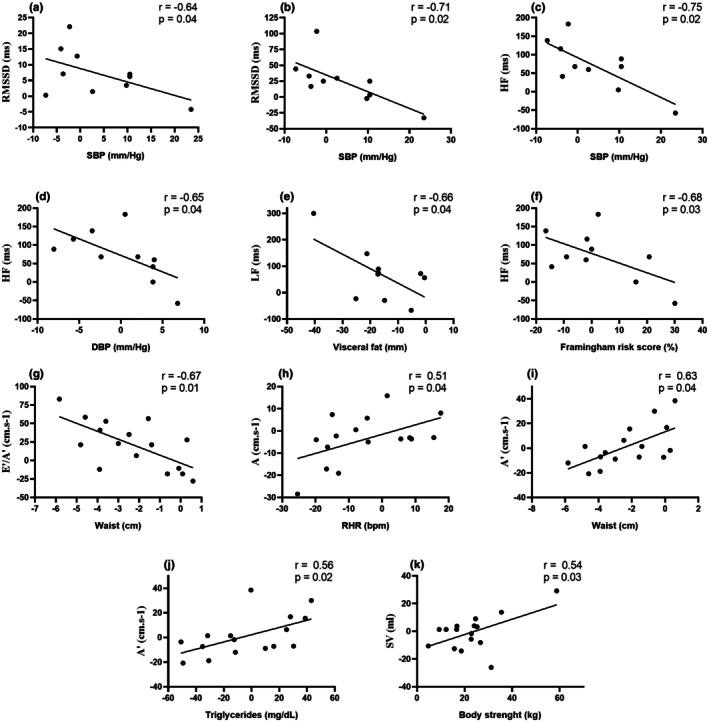
Significant Δ% correlations between HRV and echocardiogram variables with metabolic and functional markers. (a–f) *n* = 10. (g–k) *n* = 16. A peak velocity of late diastolic filling. E'/A', ratio of early and late diastolic myocardial velocities; HF, high frequency; LF, low frequency; RMSSD, the root mean square of the squares of the differences between successive; SDNN, NN interval standard deviation; SV, stroke volume.

Correlation analyses between Δ% changes in HRV, echocardiographic, metabolic, and functional variables in the CTG (Figure 2a–g) revealed significant negative associations between SBP and RMSSD, SDNN, and HF; DBP and HF; VF and LF; Framingham risk score and HF; and WC and ratio of early and late diastolic myocardial velocities (E′/A′). Positive correlations (Figure 2h–k) were observed between peak velocity of late diastolic filling (A) with resting RHR, WC, and triglycerides, as well as between stroke volume (SV) with muscular strength. Further analyses of correlations between HRV and echocardiographic variables (Tables S1 and S2) indicated significant negative associations between A with RMSSD, SDNN, and LF; and LF/HF with peak velocity of early diastolic filling (E') and E'/A'. Conversely, significant positive correlations were observed between the ratio of peak early and late diastolic filling (E/A) with RMSSD and SDNN.

## 4 DISCUSSION

The present study evaluated the effects of 24 weeks of CT on abdominal adiposity, HRV and functional and structural echocardiographic parameters in men with obesity. The main findings demonstrate that CT promotes beneficial functional adaptations in the heart, accompanied by improvements in HRV, body composition, and physical fitness variables.

In addition to the expected enhancement in ejection fraction, CT induced increases—within physiological ranges—in variables associated with myocardial relaxation and left ventricular (LV) diastolic function. These included early diastolic peak flow velocity of the mitral valve, early diastolic peak velocity of the mitral annulus, the ratio of early to late diastolic myocardial velocities, and optimization of LV filling (Douglas et al., 2019). Previous studies have reported similar improvements with aerobic training in individuals with hypertension (Leggio et al., 2014) and strength training in obese populations (Naylor et al., 2008). These results align with experimental evidence in animal models of CT‐induced myocardial adaptations (Boldt et al., 2021) and extend these findings to humans with obesity.

Stroke volume (SV) did not increase significantly despite improvements in VO_2_max, resting heart rate, and other functional cardiac parameters. Nonetheless, the CTG showed a 3.33% increase compared to a −4.58% decrease in the control group (*p* = 0.09), suggesting that a larger sample size or longer intervention duration might be required to detect statistically significant changes. Notably, a positive Δ% correlation was observed between SV and muscular strength.

The absence of significant structural cardiac changes may be attributed to the type, volume, intensity, and duration of the training protocol. Recent meta‐analyses indicate that exercise training elicits structural cardiac adaptations, with the most pronounced effects observed in predominantly aerobic and/or high‐intensity interval protocols with higher session volumes and longer durations (Morrison et al., 2023; Murray et al., 2022). In contrast, the present study employed moderate‐intensity CT. Long‐term interventions spanning years are typically associated with structural adaptations (Morrison et al., 2023; Murray et al., 2022), although shorter high‐intensity interventions can also elicit changes (Jonck et al., 2024; Saki et al., 2025).

Functional improvements in the absence of structural remodeling may result from enhanced myocyte metabolism and cardiovascular compliance, reductions in collagen deposition and fibrosis, and increases in mitochondrial biogenesis, angiogenesis, and vasodilation (Alhumaid et al., 2022). Systemic factors—including reduced subclinical inflammation and insulin resistance, improved body composition, and favorable alterations in hormonal and myokine profiles—likely contributed (Alhumaid et al., 2022; Burgess et al., 1996; Pinckard et al., 2019). In the present study, reductions in visceral fat, improvements in body composition, and decreases in DBP and resting RHR support this interpretation. Moreover, Δ% correlations indicated associations between functional variables with triglycerides, WC, and resting RHR. Reductions in inflammation and insulin resistance have also been reported in previous primary studies (Bonfante et al., 2017; Brunelli et al., 2015; Duft et al., 2017).

Enhanced autonomic modulation, as assessed by HRV, was another key contributor to cardiac functional improvements. Increases in RR interval, RMSSD, LF, and HF confirmed the effectiveness of CT in promoting autonomic benefits. These changes reflect improved heart rate oscillatory capacity, with RMSSD and HF specifically reflecting parasympathetic activity (Shaffer & Ginsberg, 2017). The increase in LF band following CT represents an interesting finding. While debates persist regarding HRV as a precise measure of autonomic modulation (Boyett et al., 2019; Malik et al., 2019), the physiological meaning of the LF band remains complex. Although it suggests both sympathetic and vagal modulation, often with some sympathetic predominance (Malik et al., 2019), recent data indicate that resting LF may primarily reflect vagal baroreflex activity (Saki et al., 2025). Thus, the observed LF increase in the present study could be attributed to an enhancement of its parasympathetic component, an interpretation consistent with concomitant increases in RMSSD and HF markers. It *is also plausible* that this LF rise represents an indirect adaptation to increased vagal activity, serving to preserve sympatho‐vagal balance, which is crucial for cardiac homeostasis (Herring et al., 2025).

Alternatively, contemporary perspectives emphasize that simultaneous coactivation of both autonomic branches is crucial for optimized cardiac function, moving beyond conventional dichotomous views (Gourine & Ackland, 2019; Herring et al., 2025). This is supported by evidence that the Central Autonomic Network contains shared regulatory areas in the brain, and cardiac innervation itself features mixed sympathetic and parasympathetic inputs with varying densities across regions (Herring et al., 2025). Indeed, even at rest, the heart receives tonic stimulation from both systems (Gourine & Ackland, 2019). During exercise, this coactivation is critical as it enhances cardiac efficiency, leading to a higher cardiac output than would be achieved by isolated sympathetic activation. This occurs through mechanisms such as extended ventricular filling time and a more powerful myocardial contraction (Gourine & Ackland, 2019). For instance, studies indicate that resting heart rate control is approximately 20% sympathetic, becomes virtually balanced during moderate exercise, and exceeds 80% sympathetic during intense exercise (Gourine & Ackland, 2019). In this context, the combined increase in the LF and HF bands observed in our study may therefore suggest a beneficial adaptation that optimizes sympatho‐vagal coactivation, contributing to enhanced flexibility and resilience in cardiac control following combined training. Furthermore, Δ% correlation analyses provided additional support for associations between cardiac functional/structural variables and autonomic markers.

Previous studies investigating the effects of exercise on autonomic modulation in populations with cardiometabolic conditions have demonstrated beneficial effects, particularly with aerobic training (Deng et al., 2024; Dias et al., 2021; Picard et al., 2021). However, studies on CT remain limited and inconsistent. Some studies reported no changes in middle‐aged women (Karavirta et al., 2013) or elderly men (Verheyden et al., 2006), whereas improvements were observed in elderly men (Karavirta et al., 2009), young obese individuals (Phoemsapthawee et al., 2019), and women with type 2 diabetes (Su et al., 2022). These discrepancies are likely attributable to differences in population characteristics (e.g., age, comorbidities) and training parameters (volume, intensity, duration). Such discrepancies likely reflect differences in participant characteristics (age, comorbidities) and training parameters (volume, intensity, duration). Alongside findings in young obese populations (Phoemsapthawee et al., 2019), the present study indicates that middle‐aged obese men can also achieve autonomic benefits from CT.

The improvements in cardiac autonomic function observed here—including HRV, vagal indices, and reductions in resting RHR and blood pressure, along with negative Δ% correlations between SBP with RMSSD, SDNN, and HF, and DBP with HF, are consistent with mechanisms involving reduced sympathetic nerve activity, enhanced baroreflex sensitivity, and improved glucose control and insulin sensitivity (Goulopoulou et al., 2010; Monahan et al., 2000). CT may promote metabolic, biochemical, hormonal, neural, functional, and body composition adaptations that support increased vagal tone (Albinet et al., 2010).

Although weight loss is commonly associated with improved parasympathetic modulation (Facchini et al., 2003; Straznicky et al., 2010), the present study observed improvements in parasympathetic indices without significant reductions in body weight. Weight loss typically enhances insulin sensitivity, lipid profile, sympathoinhibition, spontaneous baroreflex function, and reduces norepinephrine spillover (Rissanen et al., 2001). Importantly, some of these adaptations may occur even in the absence of weight loss (Bonfante et al., 2017; Brunelli et al., 2015; Duft et al., 2017). Improvements in HRV indices in the present study may be partially explained by reductions in visceral fat, as abdominal adiposity is strongly associated with sympathetic overactivity and impaired vagal modulation (Straznicky et al., 2010). Negative Δ% correlations between LF and visceral fat, and HF with Framingham risk score, further support the link between metabolic improvement and autonomic function.

Regarding Δ% correlations between HRV and echocardiographic variables, positive associations were observed between the ratio of peak early to late diastolic filling with RMSSD and SDNN, whereas negative correlations were found between the ratio of early to late diastolic myocardial velocities with LF/HF. These findings indicate that improvements in diastolic relaxation and myocardial filling patterns are associated with parasympathetic activity and improved sympathovagal balance. Maintenance or reduction of late diastolic filling combined with increased early diastolic filling induced by CT likely underpins these results, as corroborated by pre‐post comparisons and inverse correlations between peak late diastolic filling velocity with RMSSD, SDNN, and LF. Collectively, these relationships emphasize the close interplay between autonomic regulation and functional and structural myocardial adaptations (Grassi et al., 2019; Lembo et al., 2024; Shaffer & Ginsberg, 2017).

The present study has some limitations. The relatively small sample size, while consistent with human experimental protocols, may have reduced statistical power, particularly for HRV analyses. Technical issues also contributed to lower numbers of participants undergoing echocardiographic, abdominal ultrasound, and HRV assessments compared to the primary study. Finally, some results were significant only based on Δ% changes; these findings should be interpreted with caution. Nonetheless, observed statistical trends (*p* = 0.06–0.09) in post hoc tests, together with effect size calculations, can minimize this context.

## 5 CONCLUSION

Twenty‐four weeks of CT in individuals with obesity induced favorable functional cardiac adaptations and improvements in HRV. These adaptations were associated, suggesting an important interrelationship between these markers in response to CT. These changes were associated with reductions in visceral fat, improvements in body composition, and enhanced aerobic and strength performance. Collectively, these findings reinforce current exercise recommendations for cardiovascular health promotion in obese populations.

## FUNDING INFORMATION

The study was supported by São Paulo State Research Support Foundation (FAPESP)–São Paulo/Brazil–Regular Research Grants–Process: 11/09446‐6; National Council for Scientific and Technological Development (CNPQ)–Brazil–Grants process 476209/2011‐0 and 476374/2013‐8.

## CONFLICT OF INTEREST STATEMENT

The authors declare no conflicts of interest.

## ETHICS STATEMENT

All participants signed informed consent before the study. All procedures were approved by the Research Ethics Committee of the Universidade Estadual de Campinas (process number: 1278/2011).

## Supporting information


**Table S1.** Correlations of HRV with structural variables of the Echocardiogram.
**Table S2.** Correlations of HRV with functional variables from echocardiography.

## Data Availability

Data are available on request from the authors.
