# Supplementary material for: Effects of combined training on heart rate variability and cardiac function and structure in individuals with grade 1 obesity
Source: Physiol Rep. 2026 Feb 24;14(4):e70779. doi: 10.14814/phy2.70779 (PMC12932317; doi:10.14814/phy2.70779)
Supplement: Supplementary file 1 — Table S1. Correlations of HRV with structural variables of the Echocardiogram. Table S2. Correlations of HRV with functional variables from echocardiography. [file PHY2-14-e70779-s001.docx]

|  | **LAD** | **IS** | **PWT** | **RWT** | **LVM** | **LVMI** | **CO** | **LVESV** | **LVEDV** |
| --- | --- | --- | --- | --- | --- | --- | --- | --- | --- |
| **RR** | r,0228 | r,0181 | r,1708 | r,1625 | r,1893 | r,1969 | r-,2743 | r,1699 | r,0420 |
|  | p=,950 | p=,960 | p=,637 | p=,654 | p=,600 | p=,586 | p=,443 | p=,639 | p=,908 |
| **RMSSD** | r-,4383 | r-,3102 | r-,0323 | r-,0941 | r,0620 | r,0767 | r-,1885 | r,5553 | r,1312 |
|  | p=,205 | p=,383 | p=,929 | p=,796 | p=,865 | p=,833 | p=,602 | p=,096 | p=,718 |
| **SDNN** | r-,4078  p=,242 | r-,3681 | r-,0968 | r-,1399 | r,0392 | r,0346 | r-,1715 | r,5234 | r,1898 |
|  |  | p=,295 | p=,790 | p=,700 | p=,914 | p=,924 | p=,636 | p=,121 | p=,599 |
| **LF** | r-,2095  p=,561 | r-,2321 | r,1609 | r,0949 | r,1559 | r,1810 | r-,2171 | r,6225 | r-,0502 |
|  |  | p=,519 | p=,657 | p=,794 | p=,667 | p=,617 | p=,547 | p=,055 | p=,890 |
| **HF** | r-,3840  p=,273 | r-,1119 | r,0587 | r,0443 | r,1266 | r,1180 | r-,3831 | r,5635 | r,1070 |
|  |  | p=,758 | p=,872 | p=,903 | p=,727 | p=,745 | p=,274 | p=,090 | p=,769 |
| **LF/HF** | r,1329  p=,714 | r,0820 | r,0429 | r,1273 | r-,0121 | r-,0034 | r-,2713 | r-,0572 | r-,4834 |
|  |  | p=,822 | p=,906 | p=,726 | p=,974 | p=,993 | p=,448 | p=,875 | p=,157 |

**Supplementary Table 1. Correlations of HRV with structural variables of the Echocardiogram**

No significant correlations were observed. HF – High frequency. LF- low frequency. LF/HF- LF/HF ratio. IS = Interventricular septum. LAD = Left atrium diameter. LVED = Left ventricular end-diastolic volume. LVESV = Left ventricular end-sistolic volume. LVM = Left ventricular mass. LVMI = Left ventricular mass index. PWT = Posterior wall thickness. RRi mean- RR interval mean. RMSSD- the root mean square of the squares of the differences between successive R-Ri. RWT = Relative wall thickness. SDNN- NN interval standard deviation.

**Supplementary Table 2. Correlations of HRV with functional variables from echocardiography**

|  | **E/A** | **S'** | **E'** | **A'** | **E** | **A** | **E'/A'** | **EF%** | **SV** | **E/E'** |
| --- | --- | --- | --- | --- | --- | --- | --- | --- | --- | --- |
| **RR** | r,3690 | r-,2564 | r,6120 | r-,3003 | r,3341 | r-,3269 | r,6208 | r-,2442 | r-,0739 | r,1088 |
|  | p=,308 | p=,475 | p=,060 | p=,399 | p=,345 | p=,357 | p=,055 | p=,497 | p=,839 | p=,765 |
| **RMSSD** | **r,6436** | r-,2226 | r,0329 | r,5272 | r,0051 | **r-,7141** | r-,2111 | r-,1923 | r-,2209 | r,1536 |
|  | **p=,045** | p=,536 | p=,928 | p=,117 | p=,989 | **p=,02** | p=,558 | p=,596 | p=,540 | p=,672 |
| **SDNN** | **r,6680** | r-,0948 | r,2979 | r,3242 | r,0395 | **r-,7104** | r,0972 | r-,1312 | r-,1301 | r,1303 |
|  | **p=,035** | p=,794 | p=,403 | p=,361 | p=,914 | **p=,021** | p=,789 | p=,718 | p=,720 | p=,720 |
| **LF** | r,5968 | r-,0111 | r,0591 | r,4372 | r,0363 | **r-,6453** | r-,1993 | r-,1631 | r-,4495 | r,1173 |
|  | p=,069 | p=,976 | p=,871 | p=,206 | p=,921 | **p=,044** | p=,581 | p=,653 | p=,192 | p=,747 |
| **HF** | r-,1510 | r-,2039 | r,3115 | r,3383 | r-,2505 | r,0345 | r,1218 | r-,6129 | r-,2418 | r-,0773 |
|  | p=,677 | p=,572 | p=,381 | p=,339 | p=,485 | p=,925 | p=,738 | p=,070 | p=,501 | p=,832 |
| **LF/HF** | r,1321 | r-,2337 | **r-,7068** | r,1663 | r,0603 | r-,0844 | **r-,6372** | r-,0380 | r-,5027 | r,4521 |
|  | p=,716 | p=,516 | **p=,022** | p=,646 | p=,869 | p=,817 | **p=,048** | p=,917 | p=,139 | p=,190 |

Significant correlations are indicated in bold. A - peak velocity of late diastolic filling. A’ - late diastolic myocardial velocities. CO – Cardiac output; E - peak velocity of early diastolic filling. E’ – early diastolic myocardial velocities. E/A – ratio of peak early and late diastolic filling. E’/A’ – ratio of early and late diastolic myocardial velocities. E/E’ – ratio of peak early inflow velocity and longitudinal peak early diastolic velocities. EF - Ejection Fraction. HF – High frequency. LF- low frequency. LF/HF- LF/HF ratio. RRi mean- RR interval mean. RMSSD- the root mean square of the squares of the differences between successive R-Ri. S’ – systolic myocardial velocity. SDNN- NN interval standard deviation. SV – Stroke volume.
